# To what extent AstraZeneca ChAdOx1 nCoV-19 vaccine is safe and effective? Rapid systematic review

**DOI:** 10.1186/s43168-021-00109-3

**Published:** 2022-01-28

**Authors:** Aliae A. R. Mohamed Hussein, Islam H. Ibrahim, Islam A. Mahmoud, Marwa Amary, Reem Sayad

**Affiliations:** 1 Pulmonology, Chest Department, Assiut Faculty of Medicine, Assiut, Egypt; 2 Assiut Research Team (ART), Assiut, 71515 Egypt; 3 Faculty of Medicine, Assiut University, Assiut, Egypt

## Abstract

Until now, there are more than two hundred million confirmed cases of COVID-19 including more than seven million deaths. Clinical trials of all three vaccines authorized for use in the UK (Pfizer–BioNTech, Oxford–AstraZeneca, and Moderna) have reported high vaccine efficacy. This rapid systematic review was initiated because no systematic review had been conducted to determine the safety and efficacy of AstraZeneca ChAdOx1 nCoV-19 vaccine. Evidence acquisition: A systematic search in the following platforms: PubMed, Google Scholar, Scopus, WOS, and MEDLINE databases for all articles in the English language regarding safety and efficacy of SARS-CoV-2 vaccine ChAdOx1 nCoV-19 was performed. Papers published up to end of June were included. Evidence synthesis: Out of 477 retrieved articles, fifteen are included. All the selected articles are concerned with evaluation of AstraZeneca ChAdOx1 nCoV-19 vaccine. Three of them discussed the effectiveness of ChAdOx1 nCoV-19 vaccine, while thirteen (one is common with the group of the effectiveness) measured the adverse effects associated with the vaccine. Because thrombosis was recorded as a serious adverse effect developed after ChAdOx1 nCoV-19 vaccination, it was emphasized in a special group to be analyzed separately. In conclusion: the main message of selected papers was that the value of ChAdOx1 nCoV-19 vaccination to provide critical protection should be considered higher compared to the significant worldwide burden of the emerging COVID-19 infection. No causal relations were found to link cases- having thrombotic adverse reactions to the vaccine.

**Supplementary Information:**

The online version contains supplementary material available at 10.1186/s43168-021-00109-3.

## Introduction

In early December 2019, the Chinese Center of Disease Control reported that SARS-CoV-2 infection is the cause of the outbreak that started in Wuhan City [1]. SARS-CoV-2 virus is the third member of coronaviruses that causes epidemics in human history following SARS-COV and MERS. It is highly infectious and can spread globally and rapidly [2]. Until now, there are more than two hundred million confirmed cases of COVID-19 including more than seven million deaths [3].

Vaccines mimic the virus—or part of the virus—so they can protect against stimulation of the immune system to produce antibodies. Their safety standards must be higher than other medicines as they are used for the prevention of infectious diseases in healthy people and reduction of morbidity and mortality without long-lasting effects [4, 5].

For that reason, scientists are in a race with time to discover new vaccines against COVID-19. There are more than 170 candidate vaccines that are now being followed up by the World Health Organization (WHO) [6]. The first COVID-19 vaccines were approved shortly after the initial phase 3 safety and efficacy studies [7]. Clinical trials of all three vaccines authorized for use in the UK (Pfizer–BioNTech, Oxford–AstraZeneca, and Moderna) have reported high vaccine efficacy [8–10].

Large post-licensing epidemiological studies are needed to complement the results of pre-licensing trials to estimate the efficacy of these vaccines at the population level in real-world conditions, because vaccine development normally takes a very long period to confirm that vaccines are safe and effective before they are used.

This rapid systematic review was initiated because no systematic review had been conducted to determine the safety and efficacy of AstraZeneca ChAdOx1 nCoV-19 vaccine especially after publishing a number of case series which revealed serious adverse effects associated with the vaccine such as life-threatening thrombocytopenic thrombosis.

## Methods

### Study design

The study was designed as a systematic review according to PRISMA guidelines [11]. All steps of this study were pre-specified, and the protocol was registered on Clinicaltrial.gov: NCT05060861.

### Search strategy

On May 22, 2021, we searched PubMed, Google Scholar, Scopus, WOS, and MEDLINE databases for all articles in English regarding the safety and efficacy of the SARS-CoV-2 vaccine ChAdOx1 nCoV-19. The search strategy can be retrieved in supplementary digital material 1. Materials available as gray literature were followed and searched in pre-print platforms (MedRxiv, BoiRxiv), protocols, WHO reports, conference posters, thesis, or trial registers in ClinicalTrial.gov.

### Study selection

Two authors (I.A.M and M.A) independently completed all searches and removed all duplicate records. We selected the articles based on titles and abstracts. The second and last screening stage was performed by two authors (I.A.M and M.A), and discrepancies and doubts were solved by a consensus with two more authors (R.S, I.H.I). We critically appraised the full text of each study that was included if respected one of the following inclusion criteria: (1) P: volunteers (aged 18 years old or more), (2) I: ChAdOx1 nCoV-19 vaccine, (3) C: any comparator vaccine 4) O: Efficacy and Safety, (5) study design: Randomized controlled trials, retrospective studies, cohort, case-control, case series, survey, and recommendation, (6) Language: only English

### Data extraction

A data extraction form was created in word. Data were extracted by three authors (R.S., I.A.M. and M.A.) comprising the following data (if applicable): (1) study name (author/year), (2) study design, (3) study period, (4) setting (institute, city, and country), (5) study protocol number, (6) aim of study, (7) main and secondary outcome, (8) target population, (9) main age of study population, (10) classification of population according to gender, (11) sample size, (12) dose, (13) method of evaluation, and (14) conclusion

### Risk of bias assessment

We did not appraise the quality of included studies due to urgency and need of rapid appraisal of published data in this topic.

### Statistical analyses

The statistical analysis was performed using open meta-analyst software [12–14]. Dichotomous and continuous data were pooled as untransformed proportion (PR) and standardized mean difference (SMD), respectively, in a random-effects model with 95% confidence interval (CI). Heterogeneity was assessed by observation of the graphs on forest plots and measured by chi-square test and I-square tests for the degree of the heterogeneity. Between studies, significant heterogeneity was defined as a chi-square test with *p*<0.1 and *I*^2^ tests >50% [15]. We considered the endpoints statistically significant with *p* value <0.05. Irrespective of the between-study heterogeneity, subgroup analysis was done for all efficacy endpoints based on the method by which the efficacy was measured in the included studies, and the adverse events were measured depending on the number of cases developed these adverse events after vaccination.

## Results and evidence synthesis

**Fig. 1:**
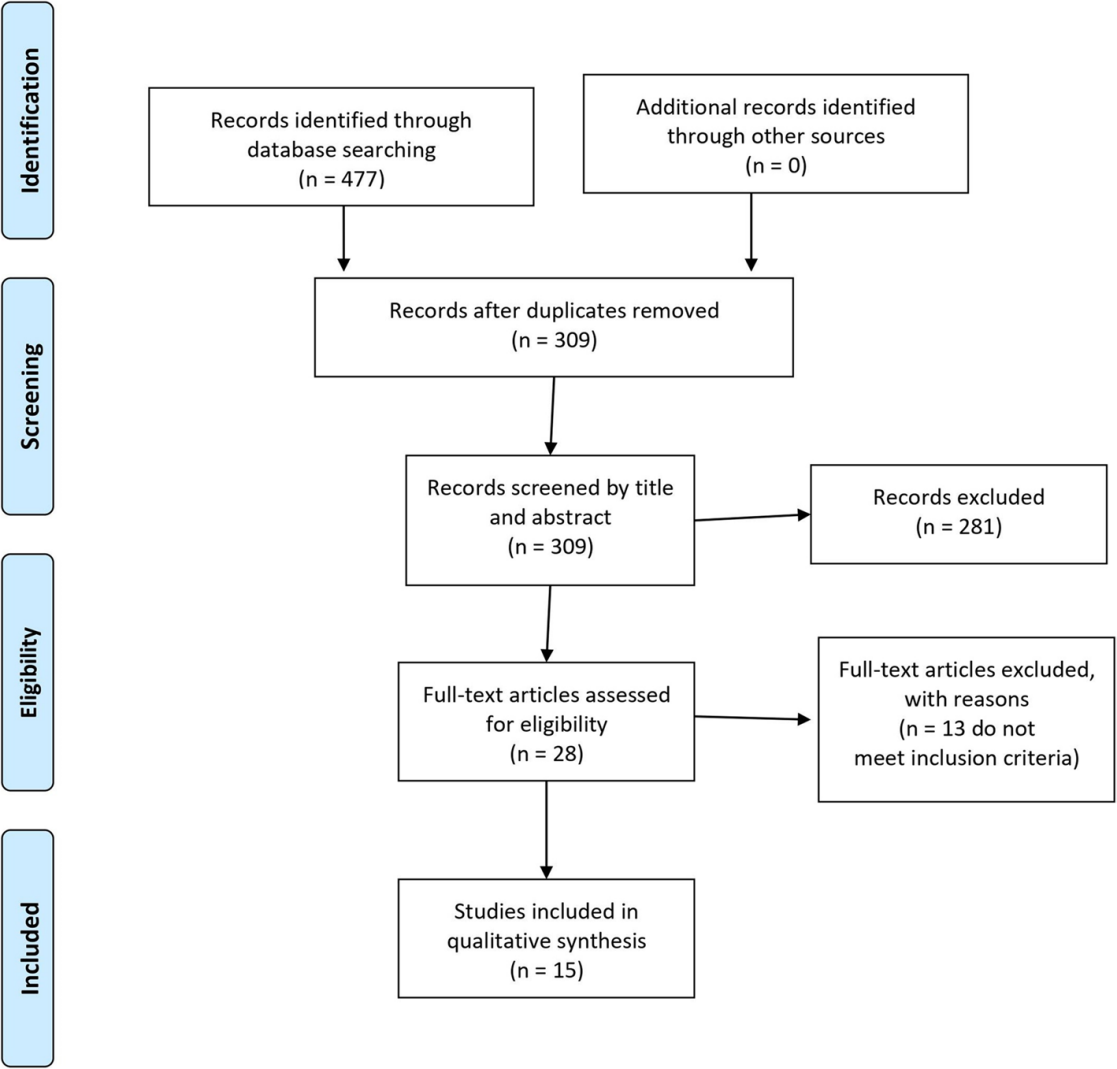
PRISMA Flow Diagram of the Present Systematic Review

Out of 477 retrieved articles, fifteen are included [16–30]. Figure 1 provides all details about the study selection process. All the selected articles are concerned with the evaluation of the AstraZeneca ChAdOx1 nCoV-19 vaccine. Three of them are concerned with the effectiveness of the ChAdOx1 nCoV-19 vaccine [18, 23, 29], while thirteen (one is common with the group of the effectiveness) consider the adverse effects associated with the vaccine [16, 17, 19–28, 30]. Because thrombosis is a serious adverse effect developed after ChAdOx1 nCoV-19 vaccination, it was placed in a special group to be analyzed separately [16, 20, 24–28, 30] (Table 1).

**Table 1:** Summary of included studies

	Study ID	Title	Aim of the study	Country involved	Duration of study	Study design	Method of evaluation	Study population or target population	Conclusion
1	Bae et al., 2021	Adverse Reactions Following the First Dose of ChAdOx1 nCoV-19 Vaccine and BNT162b2 Vaccine for Healthcare Workers in South Korea	To report the adverse reactions following the first dose of two types of vaccines against coronavirus disease 2019 (COVID-19) in healthcare workers (HCWs) in South Korea.	South Korea	March 5 and March 26, 2021.	Cross-sectional	A mobile self-report questionnaire	Healthcare workers involved in general patient care	In our prospective survey, vaccine-associated adverse reactions were more commonly reported in the ChAdOx1 group than in the BNT162b2 group. Females and younger age groups experienced vaccine-associated adverse reactions more frequently.
2	Jeon et al., 2021	Adverse Events Following Immunization Associated with Coronavirus Disease 2019 Vaccination Reported in the Mobile Vaccine Adverse Events Reporting System	To investigate the adverse events following immunization (AEFIs) for COVID-19 among healthcare workers (HCWs).	Republic of Korea.	March 3 to March 22, 2021	A retrospective,single-center cohort study	The MVAERS project developed a mobile web page to systematically capture spontaneous reporting of AEFIs.	HCWs who had completed the first dose of the ChAdOx1 nCov-19 vaccine.	The AEFIs associated with the ChAdOx1 nCoV-19 vaccine were tolerable, and the use of the MVAERS was helpful in monitoring the AEFIs. The use of MVAERS will help in sharing accurate and ample information about vaccination against COVID-19.
3	Pottegård et al., 2021	Arterial events, venous thromboembolism, thrombocytopenia, and bleeding after vaccination with Oxford-AstraZeneca ChAdOx1-S in Denmark and Norway: population based cohort study	To assess rates of cardiovascular and hemostatic events in the first 28 days after vaccination with the Oxford-AstraZeneca vaccine ChAdOx1-S in Denmark and Norway and to compare them with rates observed in the general populations.	Denmark Norway	9 February 2021 to 11 March 2021	Population-based cohort study	Complete follow-up based on computerized Danish healthcare registries, with full population coverage and daily updates.	All people aged 18–65 years who received a first vaccination with ChAdOx1-S	Among recipients of ChAdOx1-S, increased rates of venous thromboembolic events, including cerebral venous thrombosis, were observed. For the remaining safety outcomes, results were largely reassuring, with slightly higher rates of thrombocytopenia/coagulation disorders and bleeding, which could be influenced by increased surveillance of vaccine recipients. The absolute risks of venous thromboembolic events were, however, small, and the findings should be interpreted in the light of the proven beneficial effects of the vaccine, the context of the given country, and the limitations to the generalizability of the study findings.
4	Kim et al., 2021	Adverse events in healthcare workers after the first dose of ChAdOx1 nCoV-19 or BNT162b2 mRNA COVID-19 vaccination: a single center experience	To investigate adverse events (AEs) of the first dose of each vaccine, any symptom was collected daily for seven days after vaccination in a tertiary hospital.	South Korea	Began on March 5, 2021, and lasted for 7 days	Prospective cohort	Symptoms were recorded using a self-report form.	Healthcare workers (HCWs)	Reported AEs were more common in recipients with ChAdOx1 nCoV-19 than in those with BNT162b2. However, most of the reported AEs were mild to moderate in severity. Sufficient explanation and preparation for expected AEs required to promote widespread vaccination.
5	Vasileiou et al., 2021	Interim findings from first-dose mass COVID-19 vaccination roll-out and COVID-19 hospital admissions in Scotland: a national prospective cohort study	To investigate the association between the mass roll-out of the first doses of these COVID-19 vaccines and hospital admissions for COVID-19.	Scotland	Dec 8, 2020, and Feb 22, 2021	Open, real-time prospective observational cohort study	Using a unique dataset ( of the Early Pandemic Evaluation and Enhanced Surveillance of COVID-19—EAVEII—database) consisting of linked vaccination, primary care, laboratory testing (from the Electronic Communication of Surveillance in Scotland (ECOSS),8, hospital admission, and mortality data	People in Scotland who were registered in Surveillance of COVID-19 EAVE II—database	People in Scotland who were registered in Surveillance of COVID-19 EAVE II—database
6	Menni et al., 2021	Vaccine side-effects and SARS-CoV-2 infection after vaccination in users of the COVID Symptom Study app in the UK: a prospective observational study	Aimed to investigate the safety and effectiveness of these vaccines in a UK community setting.	London, UK Boston, MA, USA Lund, Sweden Uppsala, Sweden	Dec 8, 2020, and March 10, 2021	A prospective observational study “COHORT”	Self-reported information related to SARS-CoV-2 infection by App was developed by health data company ZOE Global, with input from King’s College London (London, UK)	Individuals older than 18 years can sign up to the app without any restrictions. Individuals can also record information for dependents younger than 18 years.	Systemic and local side-effects after BNT162b2 and ChAdOx1 nCoV-19 vaccination occur at frequencies lower than reported in phase 3 trials. Both vaccines decrease the risk of SARS-CoV-2 infection after 12 days.
7	Bernal et al., 2021	Effectiveness of the Pfizer-BioNTech and Oxford-AstraZeneca vaccines on COVID-19 related symptoms, hospital admissions, and mortality in older adults in England: test negative case-control study	To estimate the real-world effectiveness of the Pfizer-BioNTech BNT162b2 and Oxford-AstraZeneca ChAdOx1-S vaccines against confirmed COVID-19 symptoms (including the UK variant of concern B.1.1.7), admissions to hospital, and deaths.	England	8 December 2020 and 19 February 2021	Test negative case-control study	Laboratory findings and/or based on PCR test	All adults aged 70 years or older in England (>7.5 million people) were eligible for inclusion.	Vaccination with either one dose of BNT162b2 or ChAdOx1-S was associated with a significant reduction in symptomatic COVID-19 in older adults, and with further protection against severe disease. Both vaccines showed similar effects. Protection was maintained for the duration of follow-up (>6 weeks). A second dose of BNT162b2 was associated with further protection against symptomatic disease. A clear effect of the vaccines against the B.1.1.7 variant was found.
8	Althaus et al., 2021	Antibody-mediated procoagulant platelets in SARS-CoV-2-vaccination associated immune thrombotic thrombocytopenia	To report pathological and immunological findings in 8 patients who developed vaccine-induced immune thrombotic thrombocytopenia (VITT) after administration of SARS-CoV-2 vaccine ChAdOx1 nCoV-19.	London, UK	February 1 and April 6, 2021.	Cohort study	Clinical or laboratory findings and/or based on computed tomography, ultrasound imaging or in case of death by autopsy.	8 patients were referred to different university hospitals with neurological or hematological symptoms after vaccination with ChAdOx1 nCoV-19	Sera from VITT patients contain high titer antibodies against platelet factor 4 (PF4) (OD 2.59±0.64). PF4 antibodies in VITT patients induced significant increase in procoagulant markers (P-selectin and phosphatidylserine externalization) compared to healthy volunteers and healthy vaccinated volunteers. The generation of procoagulant platelets was PF4 and heparin dependent. We demonstrate the contribution of antibody-mediated platelet activation in the pathogenesis of VITT.
9	Folegatti et al., 2020	Safety and immunogenicity of the ChAdOx1 nCoV-19 vaccine against SARS-CoV-2: a preliminary report of a phase 1/2, single-blind, randomized controlled trial	To assess the safety, reactogenicity, and immunogenicity of a viral vectored coronavirus vaccine that expresses the spike protein of SARS-CoV-2.	Five centers in the UK	April 23 to May 21, 2020	A preliminary report of a phase 1/2, single-blind, randomized controlled trial	Humoral responses at baseline and following vaccination were assessed using a standardized total IgG ELISA against trimeric SARS-CoV-2 spike protein, a multiplexed immunoassay, three live SARS-CoV-2 neutralization assays (a 50% plaque reduction neutralization assay [PRNT50]; a microneutralization assay [MNA50, MNA80, and MNA90]; and Marburg VN), and a pseudo-virus neutralization assay. Cellular responses were assessed using an ex-vivo interferon-γ enzyme-linked immune-spot assay.	Healthy adults aged 18–55 years with no history of laboratory confirmed SARS-CoV-2 infection or of COVID-19-like symptoms	ChAdOx1 nCoV-19 showed an acceptable safety profile, and homologous boosting increased antibody responses. These results, together with the induction of both humoral and cellular immune responses, support large-scale evaluation of this candidate vaccine in an ongoing phase 3 program.
10	Greinacher et al., 2021	Thrombotic Thrombocytopenia after ChAdOx1 nCov-19 Vaccination	To assess the clinical and laboratory features of 11 patients in Germany and Austria in whom thrombosis or thrombocytopenia had developed after vaccination with ChAdOx1 nCov-19.	Germany Austria	Mid-February 2021 to March 15, 2021	Case series	Clinical and laboratory findings by using a standard enzyme-linked immunosorbent assay	Patients in Germany and Austria in whom thrombosis or thrombocytopenia had developed after vaccination with ChAdOx1 nCov-19.	Vaccination with ChAdOx1 nCov-19 can result in the rare development of immune thrombotic thrombocytopenia mediated by platelet-activating antibodies against PF4, which clinically mimics autoimmune heparin-induced thrombocytopenia.
11	Wolf et al., 2021	Thrombocytopenia and Intracranial Venous Sinus Thrombosis after “COVID-19 Vaccine AstraZeneca” Exposure	To describe the clinical manifestations and the concerning management of patients with cranial venous sinus thrombosis following first exposure to the “COVID-19 vaccine AstraZeneca”.	Germany	Began in early March 2021,	Case reports	The clinical, laboratory, and imaging findings and the results of endovascular and medicinal interventions were analyzed	Three women with intracranial venous sinus thrombosis after their first vaccination with “COVID-19 vaccine AstraZeneca” were encountered.	Early observations insinuate that the exposure to the “COVID-19 vaccine AstraZeneca” might trigger the expression of antiplatelet antibodies, resulting in a condition with thrombocytopenia and venous thrombotic events (e.g., intracranial venous sinus thrombosis). These patients’ treatment should address the thrombo-embolic manifestations, the coagulation disorder, and the underlying immunological phenomena.
12	Schultz et al., 2021	Thrombosis and Thrombocytopenia after ChAdOx1 nCoV-19 Vaccination	To report findings in five patients who presented with venous thrombosis and thrombocytopenia 7 to 10 days after receiving the first dose of the ChAdOx1 nCoV-19 adenoviral vector vaccine against coronavirus disease 2019 (COVID-19).	London, UK	March 20, 2021, to March 30, 2021,	Case Reports	Clinical and laboratory findings	Five patients who presented with venous thrombosis and thrombocytopenia 7 to 10 days after receiving the first dose of the ChAdOx1 nCoV-19	Because the five cases occurred in a population of more than 130,000 vaccinated persons, we propose that they represent a rare vaccine-related variant of spontaneous heparin-induced thrombocytopenia that we refer to as vaccine-induced immune thrombotic thrombocytopenia.
13	Scully et al., 2021	Pathologic Antibodies to Platelet Factor 4 after ChAdOx1 nCoV-19 Vaccination	To report findings in 23 patients who presented with thrombosis and thrombocytopenia 6 to 24 days after receiving the first dose of the ChAdOx1 nCoV-19 vaccine (AstraZeneca).	London, UK	N/A	Cohort study	Clinical and laboratory findings by testing for anti-PF4 antibodies was performed by means of enzyme-linked immunosorbent assays (ELISAs) at six reference laboratories in the UK and testing for anti-PF4 antibodies was performed by means of various techniques used locally for HIT testing at individual centers.	Patients were identified for the investigation of suspected vaccine-induced thrombosis and thrombocytopenia (i.e., vaccine-induced immune thrombotic thrombocytopenia, or VITT).	Vaccination against SARS-CoV-2 remains critical for control of the COVID-19 pandemic. A pathogenic PF4 dependent syndrome, unrelated to the use of heparin therapy, can occur after the administration of the ChAdOx1 nCoV-19 vaccine. Rapid identification of this rare syndrome is important because of the therapeutic implications.
14	Tiede et al., 2021	Prothrombotic immune thrombocytopenia after COVID-19 vaccine	To report five cases of prothrombotic immune thrombocytopenia after exposure to the ChAdOx1 vaccine	Germany	8th March and 4th April 2021	Consecutive single-center cohort	Clinical and laboratory findings	The patients were women between 41 and 67 years of age and presented 5 to 11 days after their first vaccination with AZD1222 (2.5×1010 particles).	an unexpected autoimmune prothrombotic disorder is described after vaccination with AZD1222. It is characterized by thrombocytopenia and anti-PF4 antibodies binding to platelets in AZD1222 dependent manner. Initial clinical experience suggests a risk of unusual and severe thromboembolic events.
15	Tobiaqy et al., 2021	Analysis of thrombotic adverse reactions of COVID-19 AstraZeneca vaccine reported to Eudra vigilance database	To identify and analyze the thrombotic adverse reactions associated with Oxford-AstraZeneca vaccine	Saudi Arabia	February 17 and March 12, 2021.	Retrospective descriptive study “COHORT”	Spontaneous reports submitted to EV database	People who were registered in the EV database in relation to COVID-19 vaccine AstraZeneca	With 17 million people having had the AstraZeneca vaccine, these are extremely rare events The EMA’s Pharmacovigilance Risk Assessment Committee (18 March 2021) concluded that the vaccine was safe, effective and the benefits outweighed the risks. Conducting further analyses based on more detailed thrombotic adverse event reports, including patients’ characteristics and comorbidities, may enable assessment of the causality with higher specificity.

### Efficacy outcomes

Three studies reported the effectiveness of the ChAdOx1 nCoV-19 vaccine. A total of 1,078,284 persons received the 1st dose and responded to the effectiveness evaluation so they are included in the analysis. The overall effect size significantly favored the effectiveness of the vaccine.

**Fig. 2:**
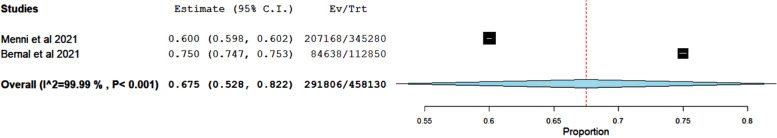
Effectiveness by decrease SARS positive after vaccination

**Fig. 3:**
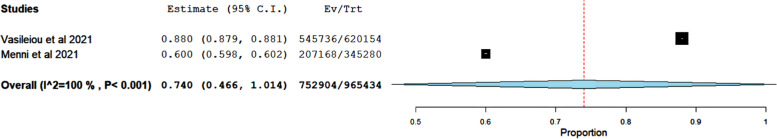
Effectiveness by decreasing hospital admission

**Fig. 4:**
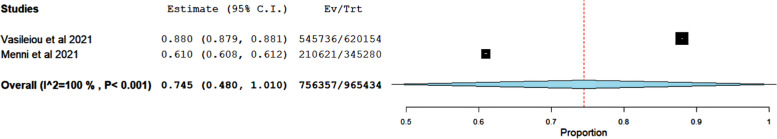
Effectiveness in elderly

- Two studies evaluated the effectiveness by decreasing SARS CoV-2 positive tests after vaccination, in which 458,130 received the 1st dose and responded to effectiveness evaluation so included in analysis. ChAdOx1 nCoV-19 vaccine significantly decreased the positive SARS-CoV-2 tests, 291806 of 458130 had negative test results after vaccination (PR= 0.675, 95% CI [0.528, 0.822], *P* < 0.001). The pooled studies were heterogeneous (chi-square *p*<0.001, *I*^2^=99.99%) (Fig. 2).
- Two studies (one study is common between 2 groups) evaluated the effectiveness by decreasing the hospitalization, in which 965,434 received the 1st dose and responded to effectiveness evaluation so included in the analysis. ChAdOx1 nCoV-19 vaccine significantly decreased hospital admission, 752,904 of 965,434 were not hospitalized after the vaccination (PR= 0.74, 95% CI [0.466, 1.014], *P* < 0.001), the pooled studies were heterogeneous (chi-square *p*<0.001, *I*^2^=99.99%) (Fig. 3).
- Two studies evaluated the effectiveness in elderly. In which 965,434 received the 1st dose and responded to effectiveness evaluation so included in the analysis. ChAdOx1 nCoV-19 vaccine significant in elderly, 756,357 of 965,434 had -ve SARS-CoV-2 test results and were not hospitalized after vaccination (PR= 0.745, 95% CI [0.480, 1.010], *P* < 0.001), the pooled studies were heterogeneous (chi-square *p*<0.001, *I*^2^= 99.999%) (Fig. 4).

### Safety outcomes

Seven studies reported the safety of the ChAdOx1 nCoV-19 vaccine. A total of 635,109 persons received the 1st dose and responded to safety evaluation so included in analysis. Of them 427,613 were female (PR=0.683, 95% CI [0.569, 0.797], *P* < 0.001). The pooled studies were heterogeneous (chi-square *p*<0.001, *I*^2^ = 99.983%).

- Analysis showed that 123,969 of 353,302 have more than one side effect, (PR=0.717, 95% CI [0.339, 1.094], *P*<0.001), the pooled studies were heterogeneous (chi-square *p*<0.001, *I*^2^ = 99.99%).
- A total number of 86,811 of older population -who were vaccinated- showed at least one side effects (PR=0.439, 95% CI [0.245, 0.633], *P*<0.001), the pooled studies were heterogeneous (chi-square *p*<0.001, *I*^2^ = 99.997%).
- Also, 36,191 of younger population—who were vaccinated—showed at least one side effects (PR=0.579, 95% CI [0.143, 1.014], *P*<0.001), the pooled studies were heterogeneous (chi-square *p*<0.001, *I*^2^ = 99.998%).
- Malaise (75.20%), headache (23.86%), fatigue (22.39%), vomiting (21.06%), chills (15.80%), joint pain (12.30%), fever (9.08%), muscle pain (8.48%), nausea (5.84%), diarrhea (2.58%), and bleeding (0.02%) are the most reported systemic side effects of ChAdOx1 nCoV-19 vaccine (Table 2).
- Local pain (11.53%), itching (2.48%), swelling (3.07%), redness (2.41%), and skin rash (0.50%) are the most reported local side effects of ChAdOx1 nCoV-19 vaccine (Table 2).
- Death was reported in only 18 of 281,272 among the vaccinated population, this is insignificant value, (PR=0.148, 95% CI [− 0.211, 0.508], *P* =0.418), the pooled studies were heterogeneous (chi-square *p* <0.028, *I*^2^= 79.161%). figures is in supplementary material 2

**Table 2:** Recorded adverse effects of ChAdOx1 nCoV-19 vaccine

	PR	95% CI	*P* value	Chi square *p* value	*I*^*2*^	Frequency
Female	0.683	0.569–0.797	< 0.001	< 0.001	99.983	427,613/635,109
Number of cases showing more than one adverse events	0.717	0.339–1.094	< 0.001	< 0.001	99.99	123,969/353,302
Yougner people with at least one adverse event	0.579	0.143–1.014	0.009	< 0.001	99.998	36,191/171,241
Older people with at least one adverse event	0.439	0.245–0.633	< 0.001	< 0.001	99.997	86,811/381,653
**Systemic side effects**						
Malaise	0.717	0.479–0.955	< 0.001	< 0.001	99.019	1156/1537
Fatigue	0.622	0.263–0.982	< 0.001	< 0.001	99.978	79,230/353,837
Headache	0.569	0.288–0.850	< 0.001	< 0.001	99.951	84,436/353,837
Muscle pain	0.516	0.035–0.998	0.036	< 0.001	99.985	29,927/352,843
Chills	0.483	0.189–0.778	0.001	< 0.001	99.952	55,985/353,837
Joint pain	0.357	0.142–0.572	0.001	< 0.001	99.908	43,522/353,837
Fever	0.346	0.109–0.582	0.004	< 0.001	99.922	32149/353837
Nausea	0.228	0.072–0.383	0.004	< 0.001	99.608	20,341/348,248
Vomiting	0.209	0.044–0.374	0.013	< 0.001	99.774	1688/8014
Diarrhea	0.154	− 0.016–0.324	0.077	< 0.001	99.879	9091/351,863
Bleeding	0.149	− 0.211–0.508	0.418	0.029	79.137	77/281,272
**Local side effects**						
Local pain	0.645	0.203–1.087	0.004	< 0.001	99.986	40,820/353,837
Itching	0.415	− 0.349–1.178	0.287	< 0.001	100	8736/351,412
Swelling	0.161	0.078–0.244	< 0.001	< 0.001	99.73	10,823/352,406
Redness	0.14	0.080–0.199	< 0.001	< 0.001	99.61	8538/353,837
Skin rash	0.031	− 0.021–0.083	0.249	< 0.001	99.65	1752/350,869
Death	0.148	− 0.211–0.508	0.418	0.028	79.161	18/281,272
**Thrombosis events**						
Percentage of thrombosis to total number of vaccinated Population	0	− 0.000–0.000	0.371	0.033	78.123	33/17,132,686
Number of cases showing more than one thrombosis events	0.515	0.281–0.749	< 0.001	< 0.001	84.009	31/83
Deep venous thrombosis	0.164	− 0.005–0.333	0.057	< 0.001	92.401	77/281,323
Thrombosis in other organs/areas	0.323	0.108–0.538	0.003	< 0.001	93.471	57/281344
Cerebral venous sinus thrombosis	0.525	0.309–0.742	< 0.001	< 0.001	96.415	46/281,347
Pulmonary embolism	0.191	0.028–0.353	0.021	< 0.001	83.733	38/281,334
Splanchnic vein thrombosis	0.103	− 0.156–0.363	0.435	0.042	75.745	23/281,275
Death	0.132	0.008–0.257	0.037	< 0.001	79.289	29/281,334

### Thrombosis outcomes

Eight studies reported thrombosis adverse events of the ChAdOx1 nCoV-19 vaccine. 281347 received the 1st dose of AstraZeneca vaccine and responded to thrombosis adverse events evaluation so included in analysis. Two hundred twenty-two thousand twenty-six of them are female (PR=0.784, 95% CI [0.755, 0.814], *P* < 0.001). The pooled studies were homogeneous (chi-square *p* = 0.404, *I*^2^= 3.415%).

- Standardized mean difference of age in the cases of thrombotic adverse events = 41.519 years old (95% CI [36.352, 46.686], *p*< 0.001), platelet count = 39.873×10^9/L^ (95% CI [27.387, 52.359], *p*< 0.001), aPTT Activated partial thromboplastin time = 29.943 s, (95% CI [25.406, 34.481], *p*< 0.001), INR peak = 1.271 (95% CI [1.152, 1.391], *p*< 0.001), fibrinogen = 1.444 g/l, (95% CI [1.015, 1.872], *p*< 0.001), and D-dimer = 33.047 mg/l (95% CI [22.703, 43.392], *p*< 0.001). The pooled studies were heterogeneous (chi-square *p* ≤ 0.001, *I*^2^= 90.204%, 95.818%, 95.724%, 85.277%, 88.658%, and 93.423%, respectively).
- The studies recorded 33 cases—of total 17,132,686 vaccinated—having thrombotic adverse reactions and this is an insignificant value (PR=0, 95% CI [− 0.000, 0.000], *P* = 0.371). The pooled studies were heterogeneous (chi-square *p* = 0.033, *I*^2^= 78.123%).
- Thirty-one cases showing more than one thrombotic adverse reaction - of total 33 who had thrombotic adverse reaction, (PR= 0.515, 95% CI [0.281, 0.749], *P* < 0.001). The pooled studies were heterogeneous (chi-square *p* < 0.001, *I*^2^= 84.009%).
- The most reported thrombotic adverse events are deep venous thrombosis (77 cases), thrombosis in other organs/areas (57 cases), cerebral venous sinus thrombosis (46 cases), pulmonary embolism (23 cases), and splanchnic vein thrombosis (23 cases) (Table 2).
- Twenty- nine of 281,334 is the number of deaths in the studies that reported thrombotic adverse reactions (PR= 0.132, 95% CI [0.008, 0.257], *P* < 0.001). The pooled studies were heterogeneous (chi-square *p* ≤ 0.001, *I*^2^= 79.289%). Figures are in supplementary material 3

## Discussion

This investigation involved a systematic review and meta-analysis of RCTs, cohorts, case series, case reports, case-control, and cross-sectional studies to summarize the efficacy and safety of the ChAdOx1 nCoV-19 vaccine. This investigation comprised 9 cohorts, 2 case reports, 1 RCTs, 1 case series, 1 case-control, and 1 cross-sectional study with a total sample size of 1,368,188 patients, 107,8284 of them were analyzed to evaluate the efficacy, and 635,184 were analyzed to evaluate the safety, with 345,280 common between two groups.

The study findings revealed that the first doses of the ChAdOx1 vaccines were associated with protection against COVID-19 admission to hospital and a decrease in the number of positive cases among the vaccinated population. A vaccine effect of 78% for protection against hospitalization and 63.7% for decreasing in the number of +ve cases among the vaccinated population. In the elderly age group, based on a pooled analysis for the vaccine, we observed vaccine efficiency of 78.3%.

The most reported systemic adverse effects associated with ChAdOx1 vaccine are malaise (75.20%), headache (23.86%), fatigue (22.39%), vomiting (21.06%), chills (15.80%), joint pain (12.30%), fever (9.08%), muscle pain (8.48%), nausea (5.84%), diarrhea (2.58%), and bleeding (0.02%). The percentage of older people with at least one adverse event (22.7%) is larger than the percentage of younger people with at least one adverse event (21.13%).

Cases with thrombotic adverse events had mean platelet count = 39.873 × 10^9/L^, (lower than normal mean) and Activated partial thromboplastin time (aPTT) = 29.943 s (within normal value). Despite these values, thrombosis also occurred. However, the recorded INR peak was 1.271, this value is lower than normal range and this may stimulate thrombosis formation. The fibrinogen level was1.444 g/l, and D-dimer 33.047 mg/l. The most reported thrombotic adverse events were deep venous thrombosis (77 cases), thrombosis in other organs/areas (57 cases), cerebral venous sinus thrombosis (46 cases), pulmonary embolism (23 cases), and splanchnic vein thrombosis (23 cases).

The increasing number of reports on rare thrombotic events after SARS-CoV-2 vaccination draw public attention and led to concerns regarding the safety of this vaccine due to the uncertainty of the origin of these undesired reactions

The limitations of this study include the small number of potential thrombotic adverse events which were contained from case reports and case series but it shouldn't be neglected because these are serious adverse events that lead to death. The quality of the included studies was not evaluated to decide the importance of the included data, due to lack of time during the pandemic.

However, this study has provided valuable information about the safety and efficacy of the ChAdOx1 vaccine from trusted databases with a large sample size and summarizes all the literature which is published until the time of searching.

Finally, the observed clinical and laboratory features of the VITT are exceptional and rare and the reported side effects cannot lead to death mostly and are relieved by medical treatment except in a few cases. Therefore, the value of COVID-19 vaccination to provide critical protection should be considered higher compared to the significant health risk of COVID-19. With the better recognition of this rare complication and the availability of efficient therapies, the risk-benefit ratio of ChAdOx1 nCoV-19 might be reconsidered further.

## Conclusions

All selected articles are based on published literature about the viral vector COVID-19 vaccine “ChAdOx1 nCoV-19.” The main message is that the value of COVID-19 vaccination ChAdOx1 nCoV-19 to provide critical protection should be considered higher compared to the significant health risk of COVID-19. Further updates are needed to follow the emerging vaccines and recognize their safety and efficacy against different variants of the novel virus

## Supplementary Information

**Additional file 1: Supplementary 1.** Modified search strategy in different databases https://docs.google.com/document/d/17NEtXgr_giWpiyVnHGdyqqs5UNZS5zGytXAQhhlgF7w/edit?usp=sharing**Supplementary 2.** Folder containing forest plots of adverse events analysis https://drive.google.com/drive/folders/17mv5gFSiRbiH6gZN16qeN_8Iix8RqmlS?usp=sharing**Supplementary 3.** Folder containing forest plots of thrombotic adverse events analysis https://drive.google.com/drive/folders/1mFN6bv2Y0atfHl0OeZKU4EmhLWFkY1Nq?usp=sharing .

## Data Availability

Data are available in the supplementary file.

## References

[1] Lu H, Stratton CW, Tang YW (2020). Outbreak of pneumonia of unknown etiology in Wuhan, China: The mystery and the miracle. J Med Virol.

[2] Huang C, Wang Y, Li X, Ren L, Zhao J, Hu Y (2020). Clinical features of patients infected with 2019 novel coronavirus in Wuhan, China. Lancet.

[3] Coronavirus W (2021). Dashboard| WHO Coronavirus (COVID-19) Dashboard with Vaccination Data.

[4] André FE (2001). The future of vaccines, immunization concepts and practice. Vaccine.

[5] Zhang C, Maruggi G, Shan H, Li J (2019). Advances in mRNA vaccines for infectious diseases. Front Immunol.

[6] Kommenda N, Hulley-Jones F (2020). Covid vaccine tracker: when will a coronavirus vaccine be ready? The Guardian.

[7] Ramasamy MN, Minassian AM, Ewer KJ, Flaxman AL, Folegatti PM, Owens DR (2020). Safety and immunogenicity of ChAdOx1 nCoV-19 vaccine administered in a prime-boost regimen in young and old adults (COV002): a single-blind, randomised, controlled, phase 2/3 trial. Lancet.

[8] Polack FP, Thomas SJ, Kitchin N, Absalon J, Gurtman A, Lockhart S, Perez JL, Marc GP, Moreira ED, Zerbini C, Bailey R (2020) Safety and efficacy of the BNT162b2 mRNA Covid-19 vaccine. New England J Med 10.1056/NEJMoa2034577 PMC7745181 33301246

[9] Voysey M, Clemens SAC, Madhi SA, Weckx LY, Folegatti PM, Aley PK (2021). Safety and efficacy of the ChAdOx1 nCoV-19 vaccine (AZD1222) against SARS-CoV-2: an interim analysis of four randomised controlled trials in Brazil, South Africa, and the UK. Lancet.

[10] Baden LR, El Sahly HM, Essink B, Kotloff K, Frey S, Novak R (2021). Efficacy and safety of the mRNA-1273 SARS-CoV-2 vaccine. New England J Med.

[11] Moher D, Liberati A, Tetzlaff J, Altman DG, Group P (2009). Preferred reporting items for systematic reviews and meta-analyses: the PRISMA statement. PLoS Med.

[12] Lau J, Antman EM, Jimenez-Silva J, Kupelnick B, Mosteller F, Chalmers TC (1992). Cumulative meta-analysis of therapeutic trials for myocardial infarction. New England J Med.

[13] Viechtbauer W (2010). Conducting meta-analyses in R with the metafor package. J Stat Software.

[14] Wallace BC, Dahabreh IJ, Trikalinos TA, Lau J, Trow P, Schmid CH (2012). Closing the gap between methodologists and end-users: R as a computational back-end. J Stat Software.

[15] Higgins JP, Thompson SG, Deeks JJ, Altman DG (2003). Measuring inconsistency in meta-analyses. BMJ.

[16] Althaus K, Möller P, Uzun G, Singh A, Beck A, Bettag M (2021). Antibody-mediated procoagulant platelets in SARS-CoV-2-vaccination associated immune thrombotic thrombocytopenia. Haematologica.

[17] Bae S, Lee YW, Lim SY, Lee J-H, Lim JS, Lee S (2021). Adverse reactions following the first dose of ChAdOx1 nCoV-19 vaccine and BNT162b2 vaccine for healthcare workers in South Korea. J Korean Med Sci.

[18] Bernal JL, Andrews N, Gower C, Robertson C, Stowe J, Tessier E (2021). Effectiveness of the Pfizer-BioNTech and Oxford-AstraZeneca vaccines on covid-19 related symptoms, hospital admissions, and mortality in older adults in England: test negative case-control study. BMJ.

[19] Folegatti PM, Ewer KJ, Aley PK, Angus B, Becker S, Belij-Rammerstorfer S (2020). Safety and immunogenicity of the ChAdOx1 nCoV-19 vaccine against SARS-CoV-2: a preliminary report of a phase 1/2, single-blind, randomised controlled trial. Lancet.

[20] Greinacher A, Thiele T, Warkentin TE, Weisser K, Kyrle PA, Eichinger S (2021). Thrombotic thrombocytopenia after ChAdOx1 nCov-19 vaccination. New England J Med.

[21] Jeon M, Kim J, Oh CE, Lee J-Y (2021). Adverse events following immunization associated with coronavirus disease 2019 vaccination reported in the mobile vaccine adverse events reporting system. J Korean Med Sci.

[22] Kim S-H, Wi YM, Yun SY, Ryu JS, Shin JM, Lee EH (2021). Adverse events in healthcare workers after the first dose of ChAdOx1 nCoV-19 or BNT162b2 mRNA COVID-19 vaccination: a single center experience. J Korean Med Sci.

[23] Menni C, Klaser K, May A, Polidori L, Capdevila J, Louca P et al (2021) Vaccine side-effects and SARS-CoV-2 infection after vaccination in users of the COVID Symptom Study app in the UK: a prospective observational study. Lancet Infect Dis 10.1016/S1473-3099(21)00224-3 PMC8078878 33930320

[24] Pottegård A, Lund LC, Karlstad Ø, Dahl J, Andersen M, Hallas J (2021). Arterial events, venous thromboembolism, thrombocytopenia, and bleeding after vaccination with Oxford-AstraZeneca ChAdOx1-S in Denmark and Norway: population based cohort study. BMJ.

[25] Schultz NH, Sørvoll IH, Michelsen AE, Munthe LA, Lund-Johansen F, Ahlen MT (2021). Thrombosis and thrombocytopenia after ChAdOx1 nCoV-19 vaccination. New England J Med.

[26] Scully M, Singh D, Lown R, Poles A, Solomon T, Levi M (2021). Pathologic antibodies to platelet factor 4 after ChAdOx1 nCoV-19 vaccination. New England J Med.

[27] Tiede A, Sachs UJ, Czwalinna A, Werwitzke S, Bikker R, Krauss JK (2021). Prothrombotic immune thrombocytopenia after COVID-19 vaccine. Blood.

[28] Tobaiqy M, Elkout H, MacLure K (2021). Analysis of Thrombotic Adverse Reactions of COVID-19 AstraZeneca Vaccine Reported to EudraVigilance Database. Vaccines.

[29] Vasileiou E, Simpson CR, Shi T, Kerr S, Agrawal U, Akbari A (2021). Interim findings from first-dose mass COVID-19 vaccination roll-out and COVID-19 hospital admissions in Scotland: a national prospective cohort study. Lancet.

[30] Wolf ME, Luz B, Niehaus L, Bhogal P, Bäzner H, Henkes H (2021). Thrombocytopenia and intracranial venous sinus thrombosis after “COVID-19 vaccine AstraZeneca” exposure. J Clin Med.

