# Supplementary material for: To what extent AstraZeneca ChAdOx1 nCoV-19 vaccine is safe and effective? Rapid systematic review
Source: Egypt J Bronchol. 2022 Jan 28;16(1):6. doi: 10.1186/s43168-021-00109-3 (PMC8795963; doi:10.1186/s43168-021-00109-3)
Supplement: Supplementary file 1 — Additional file 1: Supplementary 1. Modified search strategy in different databases https://docs.google.com/document/d/17NEtXgr_giWpiyVnHGdyqqs5UNZS5zGytXAQhhlgF7w/edit?usp=sharingSupplementary 2. Folder containing forest plots of adverse events analysis https://drive.google.com/drive/folders/17mv5gFSiRbiH6gZN16qeN_8Iix8RqmlS?usp=sharingSupplementary 3. Folder containing forest plots of thrombotic adverse events analysis https://drive.google.com/drive/folders/1mFN6bv2Y0atfHl0OeZKU4EmhLWFkY1Nq?usp=sharing . [file 43168_2021_109_MOESM1_ESM.pdf]

Review Protocol: To what extent AstraZeneca ChAdOx1 nCoV-19 vaccine is safe and effective systematic rapid review.

**Project Lead**	Dr Aliae AR. Mohamed Hussein [1,2]
**Research Team Members**	Dr Aliae AR. Mohamed Hussein [1,2]<br>aliaehussein@aun.edu.eg<br>http://orcid.org/0000-0002-7111-2195<br>Islam H. Ibrahim [2,3]<br>https://Islam.16266300@med.aun.edu.eg<br>https://orcid.org/0000-0002-3369-4964<br>Islam A. Mahmoud [2,3]<br>islam.abodeef310@gmail.com<br>https://orcid.org/0000-0002-2071-2756<br>Marwa Amary [2,3]<br>marwa.amary34@gmail.com<br>https://orcid.org/0000-0003-4333-3430<br>Reem Sayad [2,3]<br>reemoo.8527@gmail.com<br>https://orcid.org/0000-0002-4378-2714
**Date**	1/7/2021
**Institution(s)**	1- Professor of pulmonary, Assiut University hospital, Assiut 72515, Egypt.<br>2- Assiut Research Team (ART)<br>3- undergraduate at faculty of medicine, Assiut university, Egypt.

Background

to evaluate the safety and efficacy of AstraZeneca ChAdOx1 nCoV-19 vaccine

Objective

this systematic review was initiated because no systematic review had been conducted to determine the safety and efficacy AstraZeneca vaccine especially after publishing a number of case series which revealed serious adverse effects associated with the vaccine such as Thrombocytopenia

Review Question

**Full Review Question**	
safety and efficacy of AstraZeneca ChAdOx1 nCoV-19 vaccine	
**Population**	volunteers (aged 18 years old or more)
**Intervention**	chadox1 ncov-19 vaccine
**Comparison**	Any comparator
**Outcome**	Efficacy & Safety consisted of (serious) adverse events (SAEs) and adverse reactions.

Search Strategy

**Databases**
We have conducted electronic searches in the following 5 databases: PubMed, Google Scholar, Scopus, WOS and Medline with no restrictions,
**Hand Searching**
NA
**Experts or Stakeholders**
NA
**domain being studied**
New strain of corona virus (SARS-CoV2) is resulting in an increase in diseased cases around the world day after day, with more than 134 million positive cases and more than 2.9 million deaths. Due to its severity and low rate of recovery, questions have increased about the possibility of presence an effective and safe vaccine. So, we conduct this systematic rapid

review to evaluate the efficacy and safety of AstraZeneca ChAdOx1 nCoV-19 vaccine which is one of the vaccines that has been approved recently to prevent severe infection.

Eligibility Criteria

Inclusion criteria	Exclusion criteria
• Study design: Randomized controlled trials, retrospective studies, cohort, case control, case series, survey and recommendation<br>• No restriction regarding age, sex, ethnicity, place and publication Date<br>• Only English studies	• Unreliable extracted data<br>• Overlapped dataset.<br>• Only abstract available<br>• In vitro and animal studies<br>• Not English studies<br>• Case report studies

Data Extraction

A data extraction form was created in word file. Data were extracted by 2 reviewers comprising the following data if applicable: 1- Study name (author/year) 2- Study design 3- study period 4- Setting (institute, city, and country) 5- Study protocol number 6- Aim of study 7- Main outcome 8- Target population 9- Sample size 10- Dose 11- Method of evaluation 12-Preexisting conditions 13- efficacy 14- safety consisted of (serious) adverse events (SAEs) and adverse reactions.

Study Quality Assessment

We did not appraise the quality of included studies due to urgency

Data Synthesis

the synthesis of the literature is recorded in narrative and tabular format

Project Timetable

**Preparation**	5 days 16/5/ 2021-									

	20/5/ 2021									
**Conduct searches**		2 days 21&22/5 /2021								
**Pilot test eligibility criteria**			3 days 23/5/2021 – 25/5/2021							
**Ti/Ab + Full Text Selection**				15 days 26/5 – 9/6	15 days 10/6 – 23/6					
**data collection**						15 days 24/6 – 8/7				
**Revision of Data collection**							5 days 9/7- 13/7			
**Data Analysis**								7 days 14/7 – 20/7		
**Writing**									25 days	

									21/7 – 15/8	
**Final revision**										20 days 16/8 – 4/9

Research Team Member Roles

Team Member Responsible	Tasks
Dr Aliae Mohamed Hussein	Supervising and resolving any conflict for all Tasks
Miss Reem Sayad<br>Mr Islam H. Ibrahim	Preparing files and reviewing all tasks
Mr Islam A. Mahmoud<br>Miss Marwa Amary	Carrying out all tasks of the study
